# A novel variant in the *COL6A1* gene causing Ullrich congenital muscular dystrophy in a consanguineous family: a case report

**DOI:** 10.1186/s12883-021-02134-7

**Published:** 2021-03-09

**Authors:** Nirmala Dushyanthi Sirisena, U. M. Jayami Eshana Samaranayake, Osorio Lopes Abath Neto, A. Reghan Foley, B. A. P. Sajeewani Pathirana, Nilaksha Neththikumara, C. Sampath Paththinige, Pyara Rathnayake, Sandra Donkervoort, Carsten G. Bönnemann, Vajira H. W. Dissanayake

**Affiliations:** 1 Human Genetics Unit, Department of Anatomy, Faculty of Medicine, University of Colombo, Colombo 8, Sri Lanka; 2 Neuromuscular and Neurogenetic Disorders of Childhood Section, Neurogenetics Branch, NINDS, National Institutes of Health, Bethesda, USA; 3 Faculty of Medicine and Allied Sciences, Rajarata University of Sri Lanka, Saliyapura, Anuradhapura, 50008 Sri Lanka; 4 Lady Ridgeway Hospital for Children, Colombo 8, Sri Lanka

**Keywords:** Case report, *COL6A1*, Collagen type VI, Consanguineous, Myopathy, Phenotypic heterogeneity, Ullrich congenital muscular dystrophy

## Abstract

**Background:**

Collagen VI-related dystrophies are a subtype of congenital muscular dystrophy caused by pathogenic variants in *COL6A1, COL6A2* or *COL6A3* genes affecting skeletal muscles and connective tissue. The clinical phenotype ranges from the milder Bethlem myopathy to the severe Ullrich congenital muscular dystrophy (UCMD). Herein, we report the first consanguineous Sri Lankan family with two children affected with UCMD due to a novel variant in the *COL6A1* gene.

**Case presentation:**

Two sisters, aged 10-years and 7-years, presented with progressive, bilateral proximal muscle weakness. Both probands had delayed motor milestones and demonstrated difficulty in standing from a squatting position, climbing stairs and raising arms above the shoulders. Cognitive, language and social development were age appropriate. Examination showed proximal muscle weakness of the upper and lower extremities and hyperlaxity of the wrist and fingers in both with some variability in clinical severity noted between the two siblings. Serum creatine kinase levels were elevated, and electromyography showed low polyphasic motor unit potentials in the 10-year-old and myopathic features with short duration motor unit potentials with no polyphasia in the 7-year-old. Whole exome sequencing (WES) was performed and a novel, homozygous missense, likely pathogenic variant in exon 25 of *COL6A1* gene [NM_001848: c.1667G > T;NP_001839.2:p.Gly556Val] was identified in both probands. This variant was validated by Sanger sequencing in proband 1 as well as proband 2, and the parents and an unaffected sibling were found to be heterozygote carriers for the same variant.

**Conclusions:**

The findings in this family add to the expanding number of *COL6A1* variants identified and provides a better understanding of the genotype-phenotype correlations associated with UCMD.

**Supplementary Information:**

The online version contains supplementary material available at 10.1186/s12883-021-02134-7.

## Background

Collagen type VI is a microfibrillar collagen found in the extracellular matrices of muscles and connective tissue [1]. It is important in maintaining the elasticity of blood vessels and provides flexibility and strength to the skin and joints [2]. Collagen type VI consists of three main alpha chains; alpha 1, alpha 2 and alpha 3, which are encoded by *COL6A1* [OMIM#120220]*, COL6A2* [OMIM#120240] and *COL6A3* [OMIM#120250] genes, respectively [2]. Collagen VI-related dystrophies (COL6-RDs) are a subgroup of congenital muscular dystrophy resulting from heterozygous or homozygous pathogenic variants involving any of the three genes encoding the subunits of collagen type VI, and affecting skeletal muscles and connective tissue [3, 4]. The degree of muscle disorder depends on the involvement of the three alpha chains [1]. The clinical outcome may vary from a milder form known as Bethlem myopathy [MIM#158810] to a severe form known as Ullrich congenital muscular dystrophy (UCMD) [MIM#254090].

UCMD is a rare disorder with a prevalence of 0.13 per 100,000 individuals [5]. It was first described in 1930 by Otto Ullrich, as a slowly progressive disease manifesting with a combination of hypotonia, proximal joint contractures and distal joint hyperlaxity [2, 4]. Affected individuals usually have normal intelligence [1, 2, 6]. Herein, we report a consanguineous Sri Lankan family with two siblings affected with UCMD due to a novel, homozygous missense, likely pathogenic variant in the *COL6A1* gene.

## Case presentation

This study was approved by the Ethics Review Committee, Faculty of Medicine, University of Colombo, and written informed consent was obtained from the parents granting permission for genetic analyses, clinical photography and publication. Two sisters aged 7-years and 10-years were referred for genetic evaluation of progressive, proximal muscle weakness. They were born to a healthy consanguineous couple. Their firstborn child is a girl who does not have features of any muscular disorders and currently is 14 years of age. All her developmental milestones are age appropriate. Both parents are asymptomatic, and there is no family history suggestive of neuromuscular disorders. Clinical findings in the two probands are described below.

### Proband 1

**Fig. 1:**
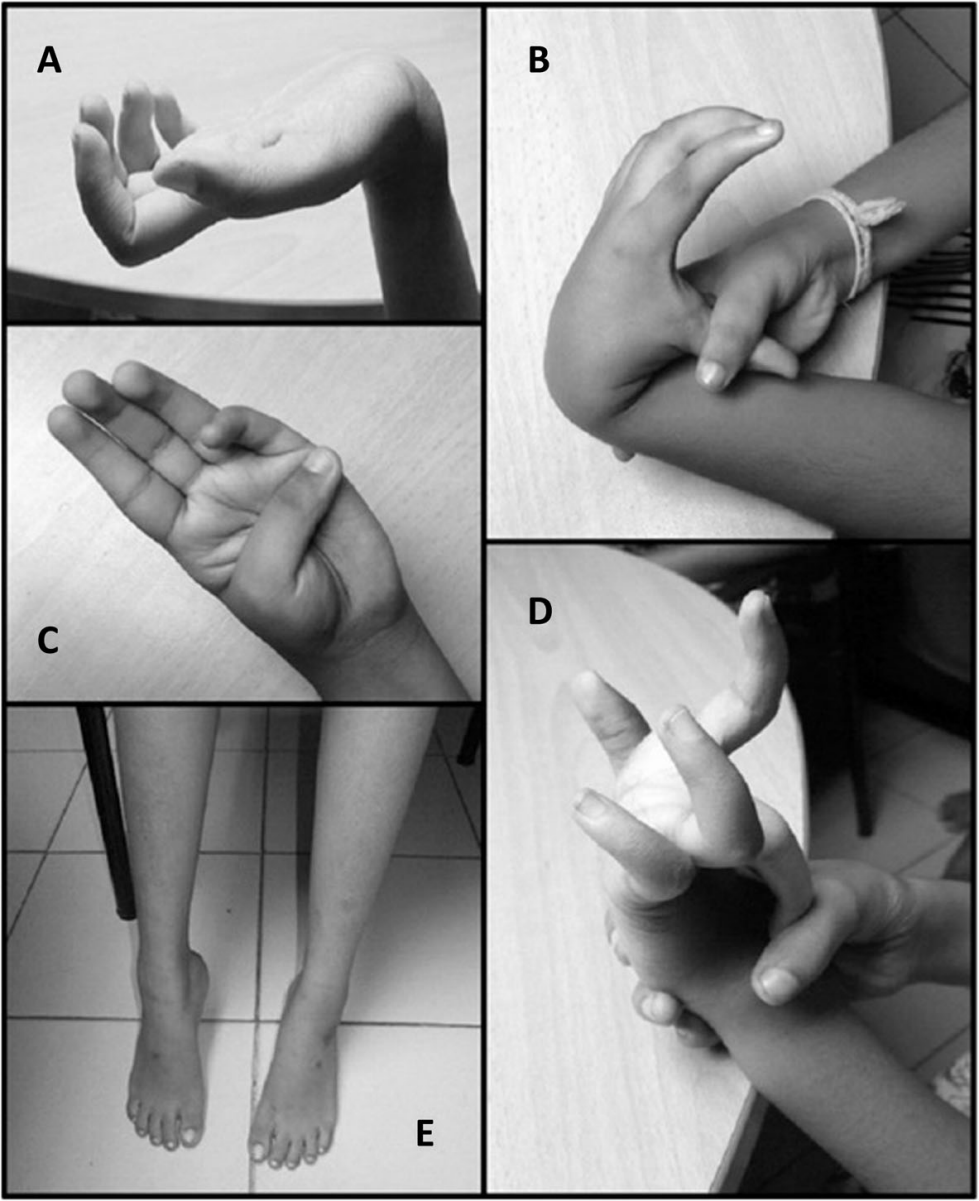
Photographs of the probands showing (**a**) hyperlaxity of the wrist joint (**b**) hyperlaxity of the wrist joint and the first metacarpophalangeal joint of the thumb (**c**) hyperlaxity of the metacarpophalangeal joint of the thumb (**d**) hyperlaxity of the metacarpophalangeal joint of the left middle finger (**e**) lower limb muscular atrophy in proband 1

Proband 1 is the second child in the family, aged 10 years. Her birth weight was 2.52 kg (10th–25th percentile), length 52 cm (50th–75th ^per^centile) and occipito-frontal circumference (OFC) 32 cm (5th percentile). Hypotonia was noted at birth. She had talipes equinovarus which was corrected with a Ponseti cast and physiotherapy. Gross motor developmental delay was noted. Muscle weakness was observed around the age of 2-years and progressively worsened with increasing age. She had bilateral proximal muscle weakness affecting the upper and lower limbs and resulting in frequent falls. She demonstrated difficulty in standing up from a squatting position, climbing stairs and raising arms above the shoulders. Her cognitive, language and social development were age appropriate, with good school performance. Clinical examination showed bilateral proximal muscle weakness in both upper and lower extremities, hypotonia, and evidence of atrophy of muscles of the bilateral lower limbs. Hyperlaxity of the distal joints was noted involving the wrists and fingers [Fig. 1]. There were no joint contractures, dysmorphic features or cutaneous manifestations noted. Examinations of the cardiovascular and respiratory systems were normal. Echocardiography was normal. She had a mildly elevated serum creatine kinase (CK) level (181 U/L; reference range 24–180 U/L). Serum electrolytes, calcium, lactate and thyroid function test results were normal. Electromyography (EMG) showed myopathic abnormalities with low polyphasic motor unit potentials. Muscle biopsy was not performed due to parental preference.

### Proband 2

Proband 2 is the third child in the family, aged 7 years. Her birth weight was 2.72 kg (10th–25th percentile), length 55 cm (90th percentile) and OFC 33 cm (10th percentile). Hypotonia was noted at birth. She had delayed motor milestones. Muscle weakness was observed at the age of 4 years and was slowly progressive. She had myopathic features similar to proband 1; however, the degree of clinical severity was comparatively less. She had reduced muscle strength and hypotonia only in the lower limbs, mainly affecting the proximal muscles, with no evidence of muscle wasting. Hyperlaxity of the distal joints involving the wrists and fingers was observed [Fig. 1]. There was no evidence of facial dysmorphism or joint contractures. Cardiovascular or respiratory systems were normal. Echocardiography was normal. Serum CK level was elevated (336 U/L). The serum electrolytes, calcium, lactate and thyroid function test results were normal. EMG findings showed myopathic features with short duration motor unit potentials with no polyphasia. Muscle biopsy was not performed due to parental preference. Both probands are being followed up at a pediatric tertiary care hospital with physiotherapy and rehabilitative care.

Blood samples were collected from all family members after obtaining parental written informed consent. Whole exome sequencing (WES) was performed on a quartet (proband 1, both parents and the unaffected sibling) at the National Institutes of Health Intramural Sequencing Center using the Illumina (San Diego, CA) TruSeq Exome Enrichment Kit and Illumina HiSeq 2000 sequencing instruments. Variants were filtered for 3 different segregation scenarios [dominant (de novo), recessive (homozygous) and recessive (compound heterozygous)] using a customized Structured Query Language script with the following parameters: minimum allele frequency below 0.5% in the Exome Aggregate Consortium (ExAc), GnomAD databases and the laboratory’s aggregate exome variant database with 770 exomes; Combined Annotation Dependent Depletion score above 20; and coverage above 10 reads. The average number of reads was 32.3 in *COL6A1*, 25.5 in *COL6A2*, and 40.42 in *COL6A3*. There were 35 total variants in *COL6A1,* 6 in *COL6A2,* and 38 in *COL6A3* in proband 1. Of these, none were truncating variants. Filtering was performed to eliminate false positive calls, polymorphisms based on population variant databases, and variants with no prediction of pathogenicity (Supplementary Table 1). Of these, only one variant in *COL6A1* survived the filtering.

**Fig. 2:**
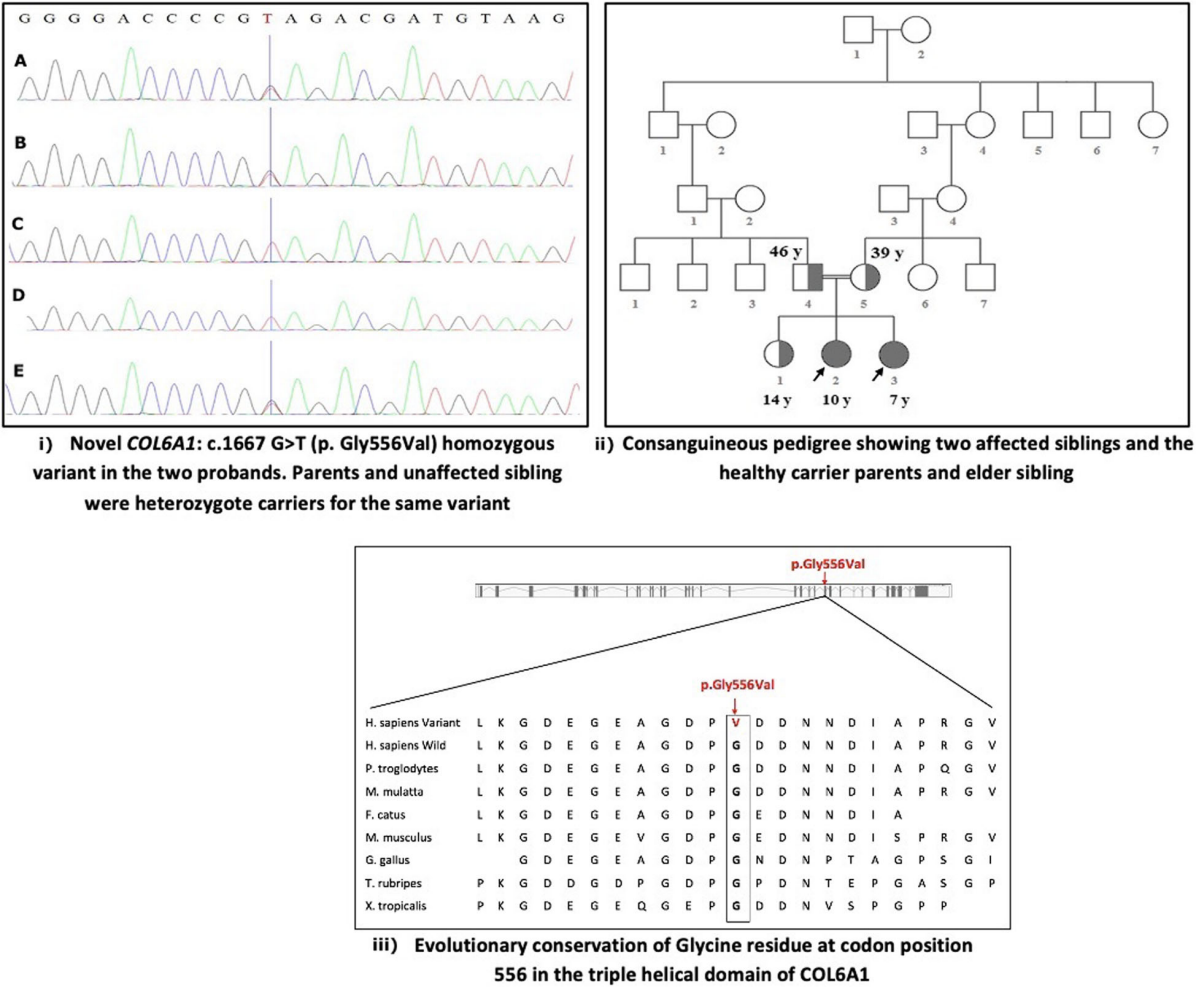
(i): Partial chromatograms from exon 25 of the *COL6A1* gene showing the heterozygous variant [NM_001848:c.1667 G > T;NP_001839.2:p.Gly556Val] in the mother (**a**), father (**b**), unaffected sibling (**e**) and homozygous variant in proband 1 (**c**) and proband 2 (**d**) (ii): Consanguineous pedigree showing the two affected siblings and the unaffected carrier parents and elder sibling (iii) Schematic diagram showing the evolutionary conservation of glycine residue at codon position 556 in the triple helical domain of *COL6A1*

A novel, homozygous missense, likely pathogenic variant in exon 25 of the *COL6A1* gene [NM_001848:c.1667G > T;NP_001839.2:p.Gly556Val] was identified in proband 1. Both parents and the unaffected sibling were heterozygote carriers for the same variant [Fig. 2]. Sanger sequencing confirmed the homozygous variant in proband 2. The novel variant is located within the triple helical (TH) domain of the COL6A1 protein at a nucleotide which is highly conserved in different animal species. At the protein level, it results in a substitution of glycine by valine at position 556 [p.Gly556Val] in the TH domain, which may result in a significant alteration of the protein structure. This variant was predicted to be damaging when analyzed using function prediction tools: Mutation Taster: disease mutation; Provean: deleterious; Polyphen2: possibly damaging; and SIFT: damaging. It is a novel variant that has not been described in the scientific literature or listed in population frequency databases or clinical databases. It was also not found in the Sri Lankan aggregate exome variant database available in our laboratory. Even though functional assays have not been carried out, the location of this homozygous variant in the COL6A1 triple helical domain coupled with the clinical phenotype make a diagnosis of UCMD highly likely. The *COL6A1* variant was submitted to the LOVD database (https://databases.lovd.nl/shared/individuals/00230584).

## Discussion and conclusions

Bethlem myopathy and UCMD result from variants including small insertions, deletions, duplications [7] or base substitutions [8] in the collagen 6 genes (*COL6A1*, *COL6A2* or *COL6A3*). The finding of a novel, homozygous, likely pathogenic missense variant in *COL6A1* along with the clinical phenotype in both probands support a diagnosis of UCMD.

Similar to previously reported cases, both probands had the characteristic phenotypic features of UCMD such as congenital symptoms of hyptonia and hyperlaxity of the distal joints, delayed motor milestone development, proximal muscle weakness, and normal intelligence [Table 1]. They did not have any skin abnormalities such as keratosis pilaris or keloid formation as frequently observed in patients with UCMD [9] or current evidence of proximal joint contractures [9–11], spinal deformities/rigidity, or respiratory insufficiency [2, 3, 9–12]. The absence of these features in both probands does not argue against the diagnosis of UCMD as these features may develop over time. As reported in previous UCMD cases, the serum creatine kinase levels were normal or slightly elevated in our probands [11, 12].

**Table 1:** Summary of the features of previously published cases of Ullrich congenital muscular dystrophy and the present study

	(Present study, 2019) Sri Lanka	(Nadeau et al., 2009) United Kingdom [9]	(Bozorgmehr et al., 2013) Iran [10]	(Martoni et al., 2013) Italy [11]	(Park et al., 2014) Korea [12]	(Brinas et al., 2010) France [13]	(Pace et al., 2008) Australia [14]	(Nalini et al., 2009) India [15]
Gender	Male:Female 0:2	not recorded	Male: Female 5:1	Male:Female 2:0	Male:Female 0:1	Male: Female 24:25	Male: Female 6:2	Male: Female 8:1
Parental consanguinity	+ (2/2)	+ (3/13)	+ (6/6)	–	not recorded	+ (7/49)	not recorded	+ (7/9)
**Clinical history / symptoms**								
Prenatal reduction of fetal movements	–	not recorded	+ (2/6)	not recorded	–	not recorded	not recorded	not recorded
Congenital contractures	–	not recorded	+ (3/6)	not recorded	+	+ (2/49)	+ (3/8)	+ (8/9)
Difficulty in walking independently	+ (2/2)	+ (4/13)	+ (6/6)	+	+	+ (46/49)	+ (8/8)	+ (9/9)
Delayed motor milestones	+ (2/2)	+ (11/13)	+ (6/6)	+	+	+ (49/49)	+ (8/8)	+ (9/9)
Progressive increase in muscle weakness	+ (2/2)	+ (13/13)	+ (6/6)	+	+	+ (26/49)	+ (8/8)	+ (9/9)
**Clinical examination**								
Abnormal facies	–	not recorded	not recorded	not recorded	not recorded	not recorded	not recorded	+ (7/9)
Joint contractures	–	+ (11/13)	+ (2/6)	+	+	+ (49/49)	+ (7/8)	+ (9/9)
Bilateral proximal muscle weakness	+ (2/2)	+ (13/13)	+ (6/6)	+	+	+ (49/49)	+ (8/8)	+ (9/9)
Gait abnormality/ inability to walk independently	+ (2/2)	+ (4/13)	+ (6/6)	+	+	+ (46/49)	+ (8/8)	+ (9/9)
Spinal rigidity/scoliosis	–	+ (12/13)	+ (6/6)	–	+	+ (43/49)	+ (5/6)	+ (7/9)
Hyperlaxity of distal joints	+ (2/2)	not recorded	+ (1/6)	+	+	+ (49/49)	+ (8/8)	+ (9/9)
Absent/indistinguishable palmar creases/soft velvet palms	+ (2/2)	not recorded	not recorded	not recorded	+	not recorded	not recorded	+ (9/9)
Atrophy of limbs with or without fasciculation	+ (2/2)	not recorded	not recorded	not recorded	+	not recorded	not recorded	+ distal muscles mainly
Keloid formation	–	+ (3/13)	not recorded	not recorded	+	not recorded	+ (2/8)	not recorded
Normal intelligence	+ (2/2)	+ (13/13)	+ (6/6)	not recorded	+	not recorded	not recorded	+ (9/9)
Respiratory function abnormality	–	+ (13/13)	+ (1/6)	–	+	+ (35/49)	+ (3/8)	+ (2/9)
Follicular hyperkeratosis	–	+ (8/13)	+ (1/6)	not recorded	not recorded	+ (22/49)	+ (7/8)	not recorded
**Investigation findings**								
EMG - short polyphasic motor unit action potentials	+ (1/2)	not recorded	+ (6/6)	not recorded	Both short and long polyphasic action potentials	not recorded	not recorded	not recorded
Muscle biopsy - infiltration of adipose tissue, atrophic muscle fibers, angulated fibers	not recorded	not recorded	not recorded	not recorded	+	+ (49/49)	+ (7/8)	abnormal (9/9)
Normal or mildly elevated CPK levels (normal 0–200 U/L)	Normal (1/2) mild elevation (1/2)	Ranged from normal to three times upper limit	Mild elevation (1/6)	Normal	Normal	Normal to four times upper limit	Normal (1/8) Mild to three times upper limit (7/8)	Normal (7/9) Mild elevation (2/9)
Genetic variants involving *COL6A1* gene	Homozygous missense variant (c.1667G > T|p.Gly556Val)	Heterozygous missense variants (c.841G > A, c.850G > A) in exon 9 and (c.868G > A) in exon 10	homozygous missense variant in exon 19	Heterozygous missense variant (intron 32c.2250 + 1 and exon 33 c.2331 Ins/Dup GCCT)	Heterozygous missense variant (c.904G > A| p.Gly302Arg); homozygous silent variant (c.1095 T > C| p.Gly365=); homozygous intronic variant (c.588 + 19dupC)	Homozygous and heterozygous *COL6A1* exon 10 mutations (majority were missense variants)	Missense variant (G > T or G > A)	Possible heterozygous variant in *COL6* gene

+, present; −, absent; *CPK* Creatine kinase, *EMG* Electromyography

In proband 1, the motor milestone development was severely delayed compared to proband 2. This was a common finding in most of the previously reported studies [Table 1]. Based on the phenotypic features in the probands, it is likely that they have deficiency of the collage VI protein. Glycine substitutions in the conserved Gly-X-Y motif within the TH domain of the COL6A1 protein have previously been demonstrated to be causative of Collagen VI myopathies including UCMD [13, 14]. Immunohistochemical analyses of collagen VI in muscle tissue or in dermal fibroblasts or functional genetic assays could not be performed to confirm decreased or absent collagen VI expression.

In conclusion, this report adds to the expanding number of *COL6A1* variants identified and provides a better understanding of the genotype-phenotype correlations associated with UCMD. These findings have important implications for the clinical management of the probands and for providing accurate genetic counseling and screening of carrier relatives.

## Supplementary Information

**Additional file 1: Table S1.** List of variants found after the filtering steps.

## Data Availability

All data generated in this study are included in this published article.

## Abbreviations

COL6-RDs: Collagen VI-related dystrophies
CK: Creatine kinase
EMG: Electromyography
OFC: Occipito-frontal circumference
WES: Whole exome sequencing
UCMD: Ullrich congenital muscular dystrophy
